# The Effect of Cobalt-Sublattice Disorder on Spin Polarisation in Co_2_Fe_x_Mn_1−x_Si Heusler Alloys

**DOI:** 10.3390/ma7031473

**Published:** 2014-02-25

**Authors:** Philip J. Hasnip, Christian H. Loach, Joseph H. Smith, Matthew I. J. Probert, Daniel Gilks, James Sizeland, Leonardo Lari, James Sagar, Kenta Yoshida, Mikihiko Oogane, Atsufumi Hirohata, Vlado K. Lazarov

**Affiliations:** 1 Department of Physics, University of York, York YO10 5DD, UK; E-Mails: s1355587@sms.ed.ac.uk (C.H.L.); josephsmith2610@gmail.com (J.H.S.); matt.probert@york.ac.uk (M.I.J.P.); dg522@york.ac.uk (D.G.); js570@york.ac.uk (J.S.); leonardo.lari@york.ac.uk (L.L.); james.sagar@ucl.ac.uk (J.S.); 2 Institute of Advanced Research, Nagoya University, Chikusa-ku, Nagoya, 464-8603, Japan; E-Mail: zoruda_rider03@yahoo.co.jp; 3 Department of Applied Physics, Graduate School of Engineering, Tohoku University, Aoba-yama 6-6-05, Aramaki, Aoba-ku, Sendai 980-8579, Japan; E-Mail: oogane@mlab.apph.tohoku.ac.jp; 4 Department of Electronics, University of York, York YO10 5DD, UK; E-Mail: atsufumi.hirohata@york.ac.uk; 5 Precursory Research for Embryonic Science and Technology, Japan Science and Technology Agency, Kawaguchi, Saitama 332-0012, Japan

**Keywords:** heusler alloys, half-metals, thin films, spintronics materials

## Abstract

In this work we present a theoretical study of the effect of disorder on spin polarisation at the Fermi level, and the disorder formation energies for Co_2_Fe*_x_*Mn_1−_*_x_*Si (CFMS) alloys. The electronic calculations are based on density functional theory with a Hubbard U term. Chemical disorders studied consist of swapping Co with Fe/Mn and Co with Si; in all cases we found these are detrimental for spin polarisation, *i.e*., the spin polarisation not only decreases in magnitude, but also can change sign depending on the particular disorder. Formation energy calculation shows that Co–Si disorder has higher energies of formation in CFMS compared to Co_2_MnSi and Co_2_FeSi, with maximum values occurring for *x* in the range 0.5–0.75. Cross-sectional structural studies of reference Co_2_MnSi, Co_2_Fe_0.5_Mn_0.5_Si, and Co_2_FeSi by *Z*-contrast scanning transmission electron microscopy are in qualitative agreement with total energy calculations of the disordered structures.

## 1. Introduction

Full Co-based Heusler alloys are predicted to be half-metallic ferromagnets, *i.e.*, 100% spin-polarised materials at the Fermi level. This unique property makes them of great interest as materials for spintronics devices [1–5]. Recently they were successfully employed as electrodes in magnetic tunnelling junctions and current-perpendicular-to-plane spin valves (CPP-SVs) [3,6–8]. Co_2_MnSi (CMS) and Co_2_FeSi (CFS) have low damping constants and high Curie temperatures, both of which are important for applications in current-induced magnetisation switching [3]. Since CMS and CFS share the same crystal structure and similar lattice constants, but slightly different half-metallic band gaps and Curie temperatures [9], by tuning the elemental composition (*x*) of Co_2_Fe*_x_*Mn_1−_*_x_*Si (CFMS), it has been suggested that both the Gilbert damping constant and the half-metallic band gap of CFMS can be tailored with respect to both CMS and CFS [9]. Moreover the structural studies of CFMS have shown that relatively well-ordered L2_1_ phase of CFMS can be achieved in comparison to CMS [7], which is a likely reason for the large room-temperature magnetoresistance (MR) of 74% that has been observed in CFMS-based CPP-SVs [10]. The CFMS-based devices have already outperformed the current-perpendicular-to-plane giant magnetoresistance (CPP-GMR) structures based on CMS and Co_2_MnGe [7,11,12]. Nevertheless the theoretical 100% magnetoresistance has not yet been realised in films or devices. Theoretical studies have shown that chemical disorder has a detrimental effect on the half-metallicity of Heusler alloys [13–16]. Recently it has been shown that the integrity of the Co sublattice is crucial for the spin polarisation of Co_2_Fe_0.5_Mn_0.5_Si at the Fermi level [17], and that the Co sublattice is relatively stable. In this work we present density functional theory (DFT) + Hubbard U calculations on spin density of states for Co_2_Fe*_x_*Mn_1−_*_x_*Si for a range of Fe concentrations, *x*, to explore the effect of a disordered Co sublattice across the chemical compositions. We note that the magnetoresistance of a device depends not only on the spin polarisation at the Fermi level, but also on the electron mobility [18], which is not considered in this work.

**Figure 1.**
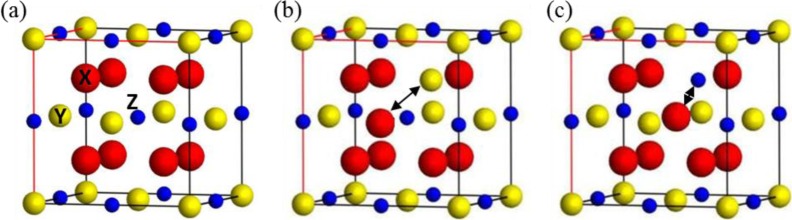
Schematic of the fully ordered L2_1_ phase, and the XY and XZ disordered phases. (**a**) L2_1_ ordered phase; (**b**) Swapping X and Y atoms; (**c**) Swapping X and Z atoms; For Co_2_(Fe/Mn)Si the X site is occupied by Co, Y by Fe/Mn and the Z site by Si. Arrows indicate swapped atoms.

Full Heusler alloys are of the form X_2_YZ, where X and Y are *d*-block elements and Z is a *p*-block element. In the ordered L2_1_-phase they possess a cubic structure with X occupying a simple-cubic sublattice (or, equivalently, two FCC sublattices) and Y and Z occupying two distinct interlocking FCC cubic sublattices (Figure 1). Grown films with cubic structure often have a degree of anti-site occupation of those cubic sites. This atomic disorder can have significant effects on the properties of the thin films, in particular the magnetisation and half-metallicity, and so it is important to understand both the nature and effects of the disorder [17,19,20] In this work we consider atomic disorder as intermixing between two elements that occupy different cubic sublattices. This “binary mixing” can be categorised according to which sites the elements are mixed between, leading to XY, YZ and XZ disorder types. For the Co-based Heuslers studied here, a previous investigation [14] showed that YZ-disordered materials retain high spin polarisation at the Fermi level, but that any XY or XZ disorder leads to a substantial degradation in the half-metallicity. Thus understanding the effects of different realisations of XY or XZ disorder in CFMS across a range of Fe/Mn concentrations is of great interest in order to understand the functionality of devices based on CFMS-type electrodes. Next we discuss the type of YZ disorder considered in this work.

## 2. Results and Discussion

In order to determine the likelihood of each type of disorder occurring, the disorder energy was computed for CFMS as a function of the Fe concentration, *x*. This disorder energy was computed for each realisation of Co-disorder as the difference between the energy per atom of the disordered cell and that of the fully ordered cell (see Table 1). Previous studies [14] on Co_2_Fe_0.5_Mn_0.5_Si showed that Co–Mn disorder has an energy of 0.11 eV/atom, which is confirmed in this work but the analogous Co–Fe disorder is found to be more favourable, with an energy of only 0.08 eV/atom. Comparing these disorder energies across the Fe concentrations (*x*) it is clear that the Co–Mn disorder energy has a maximum around *x* = 0.5, but remains relatively high throughout. In contrast the Co–Fe disorder energy is consistently lower than that of Co–Mn, and decreases monotonically as the Fe-concentration is increased, so that the Co–Fe disorder energy for CFS is only 0.06 eV/atom, almost half that of a typical Co–Mn energy. This treatment neglects the kinetic effects and mechanisms for the creation (and healing) of disorder in the Heuslers; nevertheless these energies suggest that Co–Fe disorder is much more likely to occur than Co–Mn disorder. Since typical annealing temperatures of 350–500°C correspond to *k*_B_*T* of 0.056–0.069 eV, this Co–Fe disorder will be difficult to remove by annealing, especially for high Fe concentrations. These results suggest it is desirable to have significant Mn concentrations in order to promote ordering of the Co and Fe sublattices.

Calculations of the XZ (Co–Si) disorder energies show that it is consistently larger than those of the XY disorders, regardless of the Fe concentration, with the value for *x* = 0.5 again agreeing with previous work [14]. Like the Co–Mn disorder, the XZ disorder energy reaches a maximum for intermediate Fe concentrations around 0.5≤ *x* ≤ 0.75. The absolute variation of the XZ disorder energy is the largest of all the disorders studied here at 0.03 eV/atom and total energies sufficiently large that XZ disorder is unlikely to be common in real CFMS samples.

**Figure 2.**
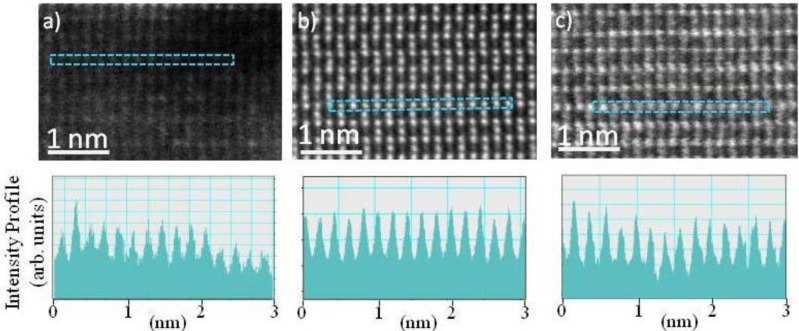
*Z*-contrast High Angle Annular Dark Field (HAADF) image of (**a**) Co_2_MnSi; (**b**) Co_2_(Fe,Mn)Si; and (**c**) Co_2_FeSi in (110) direction. Intensity profile along the X (Co site) from corresponding HAADF images.

As an indication of these finding, Figure 2 shows CMS, CF*_x_*M_1−_*_x_*S (*x*~0.5), and CFS, atomic ordering after the films were annealed at 500°C. HAADF-STEM provides a *Z*-contrast mechanism (where *Z* refers to an element’s atomic number), the relatively small difference in the atomic number between Co (X) and Mn/Fe (Y) makes quantitative analysis of the XY disorder rather challenging. However for XZ disorder contrast variation along atomic columns is much greater, and it is indicative of XZ presence (or absence) in the thin films. The very low intensity variation [from the profiles along the Co atomic columns in (110) direction, Figure 2b] from CFMS specimen (in comparison to the CMS and CFS specimens, Figure 2a,b), shows that XZ disorder is the least present in the CFMS specimen, consistent with the energy calculations presented above.

**Figure 3.**
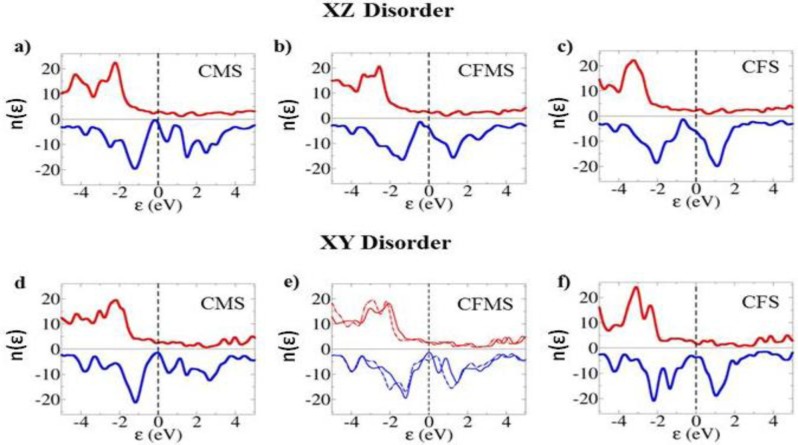
Spin density of states (SDOS) for CMS, CF_0.5_M_0.5_S, and CFS. (**a–c**) SDOS for cells with XZ disorder; *i.e*., mixing between Co (X) and Si (Z). (**d–f**) SDOS for cells exhibiting XY disorder; *i.e*., mixing between Co (X) and Mn/Fe (Y) sites. Dashed (solid) line represents case when Mn (Fe) is mixed with Co. Majority spin states are along the positive axis (red lines in colour), while minority spin states are along the negative axis (blue colour).

The effect of the disorder on the half-metallic properties of the Heusler alloys may be deduced by calculating the spin DOS, and examining the spin polarisation at the Fermi level. For a true half-metal this spin polarisation should be 100%, so that only one spin channel has states at the Fermi energy. As discussed previously, in the Co-based Heusler alloys it is the majority spin that has states at the Fermi level, and the minority spin that has a band-gap. Figure 3 illustrate the effect of the disorder on the halfmetallicity for XZ and XY disorder for CMS, CF_0.5_M_0.5_S and CFS. For both types of disorder halfmetallicity is lost, and in certain cases the spin polarisation at the Fermi level is reversed from positive to negative.

Detailed results of the spin polarisation at the Fermi level are discussed next. Since *ab initio* calculations only sample the DOS at discrete points in the Brillouin zone, it is common to smear out the states in energy using a small Gaussian energy broadening function. In this work the DOS were computed and analysed using CASTEP [21] and the OPTADOS utility program [22] with a fixed-width Gaussian smearing scheme. For small band-gaps this Fermi-broadening leads to some uncertainty in the precise value of the spin polarisation at the Fermi level, since the energy smearing is comparable to the band-gap so some apparent metallisation is inevitable. For this reason a range of smearing widths from 0.2 to 0.3 eV were applied to the DOS, to estimate the sensitivity of the results to both this smearing and the Brillouin zone sampling.

The results in Table 2 confirm that the fully ordered phases are half-metallic for all Fe concentrations. Both pure phases (CMS and CFS) are sensitive to XY and XZ disorder, with significant loss of spin polarisation at the Fermi level for all the disorders studied. The disorder in CFS (both XY and XZ) has the most dramatic effect, with more minority than majority spin states at the Fermi level leading to inverse spin polarisation.

The half-metallicities of the disordered Heusler alloys show clear trends. As illustrated in Table 2 the XY disorder of Co–Mn and Co–Fe have opposite behavior. While the Co–Mn disorder decreases the spin polarisation, and for *x* ≥ 0.25 it switches the sign of it, the spin polarisation for Co–Fe disorder decreases but stays positive for all *x* studied, except *x* = 1. Similarly the XZ disorder shows a very clear trend, and for all values of studied *x* shows negative spin polarisation, with a maximum value of −49%, except *x* = 0. In addition to spin polarisation at the Fermi level we also have calculated the net spin polarisation of the unit cells, Table 3.

In contrast to Mn, each Fe atom adds an an extra electron to the majority-spin electron, leading to an increase of 1 μ_B_ in the total magnetic moment per 16-atom unit cell for each increment of 0.25 in *x*. This trend is in accordance with the Slater-Pauling rule. The variation of the magnetisation with Fe concentration is thus smooth and monotonic, with a total change from CMS to CFS of 4 μ_B_. This simple picture does not follow for the disordered cells, where the variation is substantially less for all the disorder types. Both Co–Mn and Co–Si disorders show a trend that is the opposite of that of the pure phase, whereby the extra spin density from an Fe atom has a net contribution to the minority spin polarisation.

Finally we discuss the Co–Fe disorder, which has the lowest energy of formation and hence is of particular interest. Across the chemical compositions it is clear that in general a moderate level of spin polarisation at the Fermi level is maintained, around 30%–50%, with the notable exception of the pure CFS alloy. The disorder concentrations of 25% studied here are large, so whilst these spin polarisations are too low for device applications they do suggest that the half-metallicity of these Heusler alloys may survive in the presence of small amounts of Co–Fe order. In contrast both the Co–Mn and Co–Si disorders can completely reverse the spin polarisation at the Fermi level, which would ruin the performance of any Heusler-based device; fortunately these disorders have high energies and so are unlikely to occur in significant amounts.

## 3. Methods

To illustrate the ordering in CFMS and how it relates to CFS and CMS, films using Co–Fe–Mn–Si, Co–Mn–Si and Co–Fe–Si alloyed targets were deposited on MgO substrates. To improve the film crystallinity, annealing was performed in the sputtering chamber at 500°C for 2 h. Structural investigation was done by cross-section scanning transmission electron microscopy (STEM) using High Angle Annular Dark Field (HAADF) techniques which are *Z*-contrast sensitive on a JEOL ARM and a JEOL 2200FS microscope (JEOL Ltd., Tokyo, Japan). The cross-section specimens were prepared by conventional methods that include mechanical thinning/polishing and finishing with low energy Ar ion milling [23].

In order to study the effect of disorder on the half-metallicity of the Heusler alloys, *ab initio* electronic-structure calculations were performed using the plane-wave DFT program CASTEP [21]. Non-linear core-corrected ultrasoft pseudopotentials were used for each element, with a 650 eV plane-wave cut-off, a Brillouin zone sampling of 0.04 × 2π Å^−1^ and a Fermi level smearing of 0.2 eV. These values were chosen such that the force on a displaced atom in an ordered L2_1_ CFMS (*x* = 0.5) cell was correct to better than 1%, and the total energy was converged to within 1 meV/atom.

The effects of disorder were studied across a range of chemical compositions from pure CMS to pure CFS, starting from CMS and generating new compositions by systematically replacing Mn by Fe. The 4 Y atomic sites in the conventional 16-atom Heusler cell, allowed the study of Co_2_Fe*_x_*Mn_1−_*_x_*Si (CFMS) for *x* = 0, 0.25, 0.5, 0.75 and 1.0.

The disordered simulation systems were constructed by exchanging pairs of atoms in the ordered CFMS unit cells. Since there are four Y sites and four Z sites in the conventional cell, exchanging one Y or Z atom with an X atom corresponds to an incorrect site occupation of 25%. In this nomenclature, 50% would correspond to “full disorder,” where the correct and incorrect occupations are equally likely. For each new unit cell the lattice and atomic positions were relaxed until the maximum force on any atom was less than 0.04 eV/Å and the stress was under 0.02 GPa.

It has been shown that common semi-local approximations for exchange and correlation such as that proposed by Perdew, Burke and Ernzerhof (PBE) [24] reproduce the half-metallicity of CMS, but do not reproduce that of CFS [25]. In CFS the minority spin band-gap is closed, causing full metallicity of the Heusler; this closing of the band-gap arises from the self-interaction error in standard DFT, which raises the energy of the pseudo-atomic *d* states and causes them to lie too close to the Fermi level. This anti-site positioning of the *d* states in energy can be corrected approximately by the addition of a localising Hubbard potential of strength U into the Kohn-Sham Hamiltonian [17,25]. For this work PBE + U with a value of U = 2.1 eV was used for the *d* states of all Co, Mn and Fe sites in the ordered and disordered cells. The inclusion of U widens the minority spin-gap and recovers the half-metallic properties of the bulk, ordered Heuslers CFMS for all chemical compositions studied (0.0 ≤ *x* ≤1.0). Spin density of states (SDOS) calculations for the L2_1_ ordered phases of CMS, CFMS and CFS confirmed extremely high (99%–100%) spin polarisation at the Fermi level, with a typical minority-spin Kohn-Sham band-gap of around 1 eV. It should be noted that DFT + U is only an approximate correction for the self-interaction error, and furthermore the Kohn-Sham states do not represent the true quasiparticles of the system, so the calculated band gaps will still be inaccurate compared to experiment; nevertheless the band-gap trends computed by DFT are widely considered to be accurate.

## 4. Conclusions

*Ab initio* electronic structure calculations of various XY and XZ disordered phases of CFMS show that Co–Fe disorder is the lowest energy of the Co-disorders, sufficiently low that it may be difficult to remove by annealing. However, with the exception of CFS, the effect of Co–Fe disorder on the half-metallic property of CFMS is relatively modest, suggesting that CFMS alloys may be robust to small amounts of this disorder. In contrast Co–Mn and Co–Si disorder can have dramatic effects on the half-metallicity across all compositional ranges, but their energies are sufficiently large that they are unlikely to occur in great quantity may be easier to heal by annealing. These results may explain the high magnetoresistance experimentally observed in spin valves in CFMS films [10].

## Figures and Tables

**Table 1.** Disorder energies (eV/atom) for a range of Fe-concentrations (*x*) and Co-disorder types.

Disorder type	*x* = 0	*x* = 0.25	*x* = 0.5	*x* = 0.75	*x* = 1.0
Co–Mn (XY)	0.10(5)	0.11 (3)	0.11 (8)	0.11 (5)	–
Co–Fe (XY)	–	0.08 (5)	0.08 (2)	0.07 (1)	0.06 (1)
Co–Si (XZ)	0.16 (2)	0.17 (3)	0.19 (2)	0.19 (2)	0.18 (6)

**Table 2.** Spin polarisation at the Fermi level for Co_2_Fe*_x_*Mn_1−_*_x_*Si for a range of Fe-concentrations (*x*) and disorder types. The range of spin polarisations correspond to a range of Fermi level broadenings from 0.2 to 0.3 eV.

Disorder type	*x* = 0	*x* = 0.25	*x* = 0.5	*x* = 0.75	*x* = 1.0
Co–Mn (XY)	24% to 38%	−12% to −16%	−23% to −28%	−24% to −37%	–
Co–Fe (XY)	–	28% to 34%	29% to 43%	31% to 48%	−36% to −38%
Co–Si (XZ)	31% to 56%	−12% to −14%	−19% to −24%	−42%	−48% to −49%
Ordered	95%	99%	100%	100%	99% to 100%

**Table 3.** Total spin polarisation (magnetic moment) of Co_2_Fe*_x_*Mn_1−_*_x_*Si in μ_B_ per 4-atom formula unit, for a range of Fe-concentrations (*x*) and disorder types.

Disorder type	*x* = 0	*x* = 0.25	*x* = 0.5	*x* = 0.75	*x* = 1.0
Co–Mn (XY)	6.075	6.0	5.825	5.85	–
Co–Fe (XY)	–	5.425	5.55	5.35	5.6
Co–Si (XZ)	6.45	6.5	6.4	6.325	6.175
Ordered	5.05	5.275	5.5	5.75	6.0
